# A Photocatalytic Rotating Disc Reactor with TiO_2_ Nanowire Arrays Deposited for Industrial Wastewater Treatment

**DOI:** 10.3390/molecules22020337

**Published:** 2017-02-22

**Authors:** Fang Li, Wai Szeto, Haibao Huang, Jiantao Li, Dennis Y. C. Leung

**Affiliations:** 1 Department of Mechanical Engineering, University of Hong Kong, Hong Kong, China; lifang88@hku.hk (F.L.); u3003605@hku.hk (W.S.); huanghb6@sysu.edu.cn (H.H.); 2 School of Environmental Science and Engineering, Sun Yat-sen University, Guangzhou 510000, China; 3 Sinopec Fushun Research Institute of Petroleum and Petrochemicals (FRIPP), China Petroleum & Chemical Corporation, Fushun 113006, China

**Keywords:** TiO_2_ nanowire arrays, photocatalytic rotating reactor, wastewater treatment

## Abstract

A photocatalytic rotating disc reactor (PRD-reactor) with TiO_2_ nanowire arrays deposited on a thin Ti plate is fabricated and tested for industrial wastewater treatment. Results indicate that the PRD-reactor shows excellent decolorization capability when tested with methyl orange (>97.5%). Advanced oxidation processes (AOP), including photocatalytic oxidation and photolytic reaction, occurred during the processing. Efficiency of the AOP increases with reduction in light absorption pathlength, which enhanced the photocatalytic reaction, as well as by increasing oxygen exposure of the wastewater thin film due to the rotating disc design. It is found that, with a small dosage of hydrogen peroxide, the mineralization efficiency of industrial biodegraded wastewater can be enhanced, with a superior mineralization of >75% total organic carbon (TOC) removal. This is due to the fact that the TiO_2_ photocatalysis and hydrogen peroxide processes generate powerful oxidants (hydroxyl radicals) that can strongly improve photocatalytic oxidation efficiency. Application of this industrial wastewater treatment system is benefited from the TiO_2_ nanowire arrays, which can be fabricated by a mild solvothermal method at 80 °C and under atmospheric pressure. Similar morphologies and microstructures are found for the TiO_2_ nanowire arrays deposited on a large metal Ti disc, which makes the wastewater treatment process more practical and economical.

## 1. Introduction

Since the first research paper from Fujishima and Honda regarding photocatalytic water splitting on an anatase TiO_2_ electrode was reported [1], substantial research work has been conducted, showing that nanostructured TiO_2_ is an effective material for photocatalytic wastewater treatment [2]. This is mainly because TiO_2_ has several remarkable physicochemical properties, such as photocatalytic activity, chemical stability, nontoxicity, and low cost. Numerous researchers studied photocatalytic degradation, focused on various photocatalysts in powder form [3,4,5,6]. However, some inevitable problems, such as recyclability and secondary pollution, exist for TiO_2_ in a suspended powder form. An immobilized TiO_2_ thin film photocatalyst can address these problems well. Metal titanium is a cheap material and there is a large reserve of it on Earth. Use of titanium plates as Ti source to prepare TiO_2_ nanostructure film material is a promising preparation method, and, in this regard, highly ordered TiO_2_ nanotube arrays prepared by anodization have been reported [7,8,9,10]; however, they require a massive electrical power consumption during the fabrication process. Additionally, a number of studies also involve nanostructured TiO_2_ deposited on conductive glass through a solvothermal method, conducted at temperatures higher than 100 °C [11,12]. Li et al. [13] reported a facile solution route to deposit aligned TiO_2_ nanowires on arbitrary substrates at a temperature of 80 °C under an open atmosphere, which expands the possibility of industrial applications due to its low-cost synthesis conditions.

Photocatalytic oxidation technology is a potentially useful way to purify wastewater, owing to the powerful oxidation property of the photocatalyst to damage the molecular structure of pollutants [14], as well as to deactivate bacteria in polluted water [15]. However, industrial applications of photocatalytic technology are still limited due to the low recyclability of the photocatalyst powder and the inefficient photocatalytic reaction caused by the recombination of photo-generated electron-hole pairs [16,17]. Furthermore, light utilization efficiency also has a considerable role in photocatalytic reactions because it is the main triggering factor in the process.

The present study shows that rotating film disc design can solve the above-mentioned problems. First, light utilization efficiency of the photocatalyst, which reduces due to solution absorption, can be optimized by reducing the path length of light through the rotating disc design. The superhydrophilic characteristics of TiO_2_ make it possible to form a thin aqueous film on its surface when rotating, which can effectively increase the radiation power in order to get a high quantum efficiency [18]. Second, for this device, the upper half of the circular TiO_2_ disc is exposed to air, and the main driver for TiO_2_ photocatalytic activity is the formation of radicals, of which O_2_ serves as an important scavenger of the photogenerated carrier charge in this reaction [19]. Therefore, the upper half of the TiO_2_ disc that is exposed to air can gain a significant improvement in photocatalytic activity due to reduction of the recombination of photogenerated electron-hole pairs. On the other hand, the solution adhered to the TiO_2_ disc surface is incessantly refreshed through the rotating process, which promotes an aqueous exchange between wastewater and degraded products. The present photocatalytic rotating disc reactor (PRD-reactor) is powerful because it, not only acts under an improved photocatalysis process, but also combines with ultraviolet (UV) lamp irradiation, which involves a highly effective photolysis process for simultaneously degrading wastewater in a single treatment system.

The effects of H_2_O_2_ on photocatalytic process were investigated in detail by Fernández et al. [20], who indicated that photocatalytic degradation of pollutants could be more effective in the presence of H_2_O_2_ in appropriate concentrations. In addition, H_2_O_2_ was also studied in the inactivation of bacteria under visible light-induced photocatalytic reaction [21,22]. Here, the low concentration of H_2_O_2_ can serve as an electron acceptor in the photocatalytic process in order to produce active radicals to improve the reaction with an environmentally-friendly dosage.

## 2. Results

### 2.1. Morphologies and Microstructure

**Figure 1:**
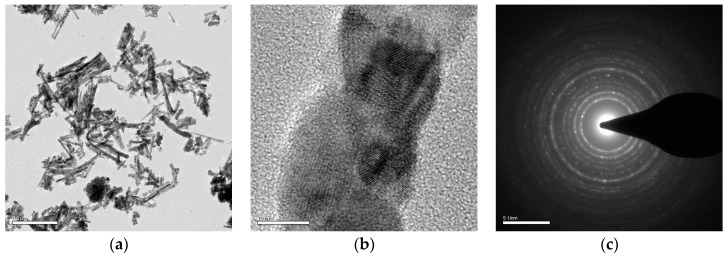
(**a**) The transmission electron microscopy (TEM) images of the scraped TiO_2_ nanowire (scale bar: 0.5 μm); (**b**) the high resolution TEM images of a part from a single TiO_2_ nanowire (scale bar: 10 nm); (**c**) selected area diffraction pattern of TiO_2_ nanowire (scale bar: 51/nm).

The transmission electron microscopy (TEM) images of the scraped fractals of TiO_2_ nanowire deposited on the Ti plate (Figure 1a) clearly show that the as-prepared samples still contain a TiO_2_ nanowire array structure. The length of the nanowires vary from 200 nm to 500 nm, while the diameters are between 15 and 25 nm. The length to diameter ratio is about 20:1, demonstrating that this synthesized material has a very large specific surface area, which benefits a higher reaction rate. The crystalline state is revealed by the high resolution transmission electron microscopy (HR-TEM) images (Figure 1b,c), which showed that, after calcination, the TiO_2_ nanowire arrays are mixed crystals, which are reported, in the literature, to possess good photocatalytic activity [23,24].

**Figure 2:**
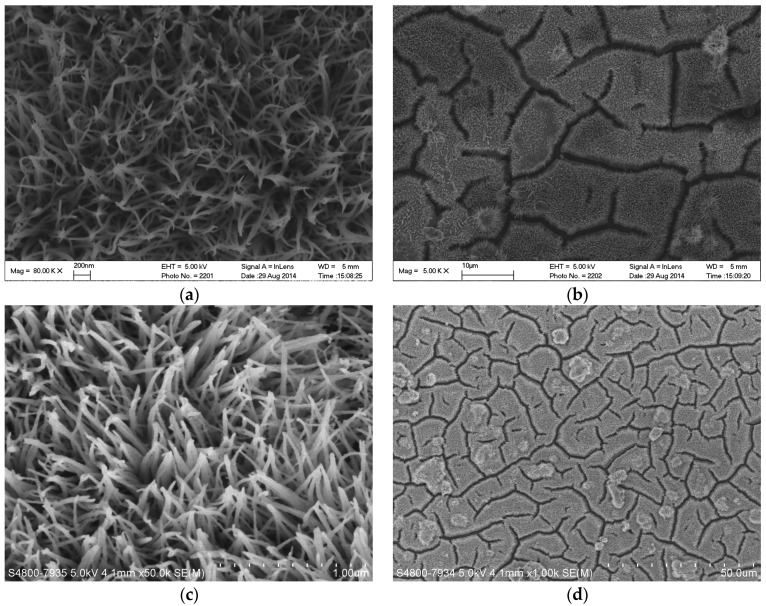
Scanning electron microscope (SEM) images of TiO_2_ nanowire arrays: the sample of rectangular plate (20 mm × 30 mm) with 0.2 mm thickness under the magnification of 80.0 k (**a**) and under the magnification of 5.0 k (**b**); the sample of circular disc (φ200 mm diameter) with 0.8 mm thickness under the magnification of 50.0 k (**c**) and under the magnification of 5.0 k (**d**).

Top-view of the scanning electron microscope (SEM) images of the TiO_2_ nanowire arrays are shown in Figure 2a,c. Clear, tight, and orderly TiO_2_ nanowire arrays were observed with very large specific surface areas. The cracks on the base, which are formed by the calcination process, were seen in the low-magnification SEM images (Figure 2b,d). Different thicknesses of titanium plates (i.e., 0.1 mm, 0.2 mm and 0.8 mm) with the same surface size (20 mm × 30 mm) were chosen to investigate the influence of substrate thickness on the formation of TiO_2_ nanowire arrays. Experimental results demonstrated that this mild preparation method is stable because the TiO_2_ nanowire arrays can be deposited successfully on all the three Ti substrates of different thicknesses with surface area of 20 mm× 30 mm. Especially, as shown in Figure 2c, an enlarged titanium plate (φ200 mm with a 0.8 mm thickness), with tight and orderly TiO_2_ nanowire arrays deposited on the surface, was also obtained through this mild preparation method, which confirmed that the method can be applied for the preparation of large-scale materials for industrial design.

**Figure 3:**
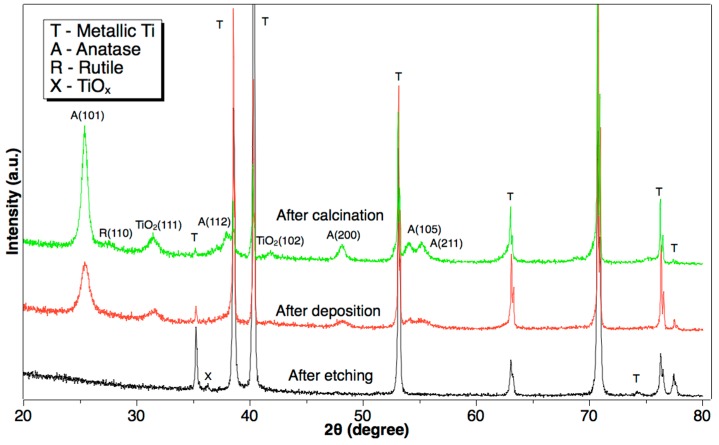
X-ray diffraction patterns of TiO_2_ obtained using metallic Ti after each step. Undefined TiO_2_ belongs to the Joint Committee on Powder Diffraction Standards (JCPDS) card 21-1236; X is TiO*_x_* with JCPDS card 43-1295. The content of the insert text box are abbreviations for each substance, which are T for metallic Ti, A for Anatase TiO_2_, R for Rutile TiO_2_, X for TiO_X_, respectively. a.u.: arbitrary units.

The influences on X-ray diffraction (XRD) of each step for the preparation of the TiO_2_ nanowire arrays are presented in Figure 3. As shown, there are only characteristic diffraction peaks of metallic Ti after the etching step; however, a less obvious peak can be observed at 36.2°, which corresponds to TiO*_x_* of the Joint Committee on Powder Diffraction Standards (JCPDS) card 43-1295. This probably means that, in the process of etching, the surface of the Ti plate was, not only cleaned in the acidic solution (removing impurities), but also was preliminarily oxidized by the F^−^-containing solution. In the process of deposition, according to the XRD pattern, anatase TiO_2_ (JSPCS card 21-1272) already formed, but with a subcrystalline structure, and the peak of TiO*_x_* (110) disappeared. After annealing at 500 °C for 2 h, the characteristic diffraction peaks of the anatase phase became much sharper; a peak of the rutile phase (110) at 27.5° even appeared. In addition, two small peaks at 31.5° and 42.0°, along with the anatase formed in the deposition step, and crystalized completely in the calcination step, which belongs to the phase (111), (102) of the other kind of TiO_2_ (JCPDS card 21-1236), respectively, without photocatalytic activity [25].

**Figure 4:**
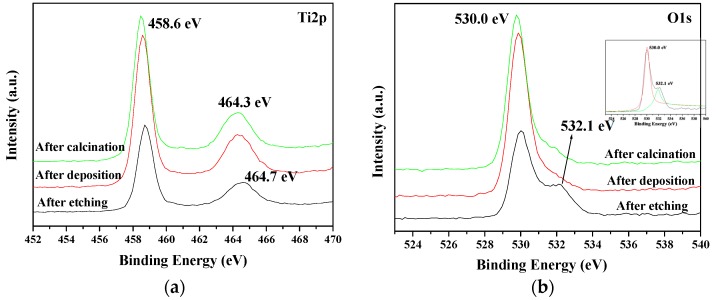
X-Ray photoelectron spectroscopy spectra of TiO_2_ nanowire arrays under different preparation stages. (**a**) Ti2p: 2p orbit of titanium; (**b**) O1s: 1s orbit of oxygen.

Figure 4 shows the X-ray photoelectron spectroscopy (XPS) of TiO_2_ nanowire arrays under different preparation stages, which helps in the investigation of the valence states and chemical composition of the sample. As shown, two peaks can be observed, which belong to Ti 2p_1/2_(~464.3 eV) and Ti 2p_2/3_(458.6 eV), respectively. The binding energy of Ti2p shifted slightly after every preparation stage, meaning that Ti-ion was formed on the surface of the Ti plate during the etching process. On the other hand, two peaks (i.e., 530.0 eV and 532.1 eV) emerged in the XPS spectra of O1s after etching the Ti plate, while the peak at 532.1 eV disappeared after the deposition. According to Hiromoto et al. [26], these two kinds of O peaks came from TiO_2_ (530.0 eV) and Ti-OH (532.1 eV), respectively. The result indicated that the Ti plate was fluorided and was preliminarily oxidized during the etching process, which promoted further oxidation to produce the TiO_2_ nanowire arrays.

### 2.2. Photoelectrochemical Performance

**Figure 5:**
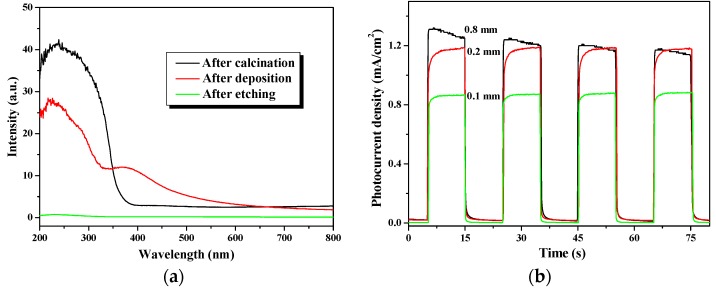
(**a**) Diffuse reflection spectrum of TiO_2_ nanowire arrays; (**b**) photocurrent of TiO_2_ nanowire arrays deposited on the Ti plate with different thicknesses.

Figure 5a shows the ultraviolet-visible (UV-vis) light absorption properties of the TiO_2_ nanowire arrays under different preparation stages. As shown, after etching of the Ti plate, no active TiO_2_ with light-response properties was formed. However, the sample after deposition revealed a few light absorption peaks, which could be attributed to the formation of anatase TiO_2_. Because the as-prepared TiO_2_ was in an unstable state and contained impurities after the deposition process, the light absorption curve was slightly unusual. After calcination, the deposited TiO_2_ was transformed into mixed phases of anatase and rutile, which have outstanding light responses in the UV region. The excellent intrinsic light absorption capability of the TiO_2_ nanowire arrays after calcination ensure the high efficiency of its photocatalytic activity.

Figure 5b shows the photocurrent densities of the TiO_2_ nanowire arrays deposited on the Ti plates with different thicknesses (i.e., 0.1, 0.2 and 0.8 mm). To test the photoelectrochemical response of the TiO_2_ nanowire arrays under light irradiation, the system was operated under a light on–off process, with a pulse of 10 s using the potentiostatic technique with a +0.5 V bias potential for an N-type semiconductor in a 0.2 M Na_2_SO_4_ solution. As shown in Figure 5b, the stronger intensity of photocurrent responses exhibited a higher separation yield of photo-generated electron-hole pairs, which led to a more efficient photocatalytic reaction (as shown later). The result indicates that the photocurrent increased with the thickness of the TiO_2_ substrate. The photocurrent density (1.19 mA/cm^2^) of the TiO_2_ nanowire arrays with a 0.2-mm thick Ti plate is significantly higher than that of the 0.1-mm thick Ti foil (0.84 mA/cm^2^). The reason for this result is that a thicker substrate, like a Ti plate with a thickness of 2 mm, is stronger than a 1-mm thick Ti foil, which is strong enough to prevent the TiO_2_ nanowire from peeling off the surface of the substrates due to bending. This implies that a thicker substrate is beneficial for a more efficient deposition and formation of TiO_2_ nanowire arrays. In consideration to the rigidity of the Ti plate to be fixed on the rotating shaft of the reactor sturdily, increasing the thickness of the substrate to 0.8 mm, and the photocurrent of 1.2 mA/cm^2^ is similar to the photocurrent density of the 0.2-mm thick Ti plate, which is good for reactors requiring high stability and reuse capabilities.

### 2.3. Photocatalytic Activity Test

#### 2.3.1. Stagnant Test

**Figure 6:**
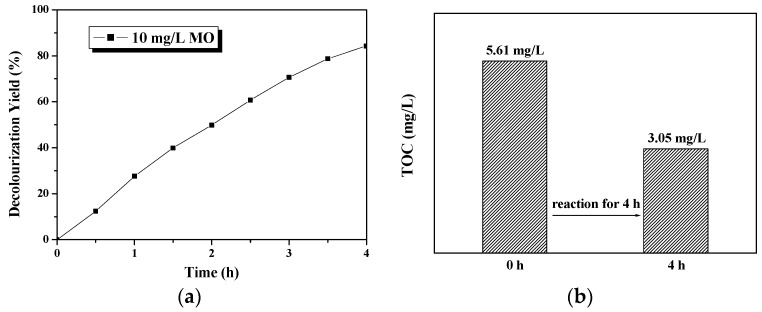
Photocatalytic activity test for methyl orange (MO). Reaction conditions: 45 mL of 10 mg/L MO as degradation target, 7 W ultraviolet (UV) lamp as light source, TiO_2_ nanowire photocatalyst deposited on 0.2-mm substrate with 20 mm × 20 mm active area immersed in the pollutant solution; the distance between light source and catalytic film is 20 mm. The reaction was conducted in a quartz beaker under magnetic stirring. (**a**) The line curve is decolorization yield changes with time; (**b**) the bars diagram represents the initial and final total organic carbon (TOC) value after the 4-h reaction.

Figure 6 reveals the stagnant test results of methyl orange decolorization using the Ti plate deposited with the photocatalyst and tested in a beaker; a decolorization yield of 84% can be obtained after 4 h of photocatalytic reaction under the irradiation of a 7 W UV light. The corresponding total organic carbon (TOC) value decreased from 5.61 mg/L to 3.05 mg/L after four hours, representing an effective mineralization rate of 46%. The stagnant test results show that the TiO_2_ nanowire arrays deposited on the Ti plate, prepared via this mild, low-temperature solvothermal method has a favorable photocatalytic activity.

**Figure 7:**
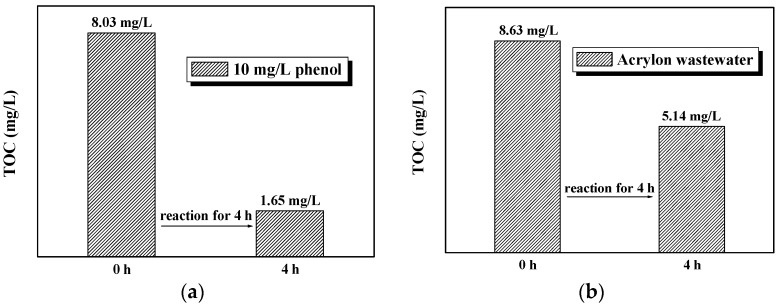
Photocatalytic activity test for (**a**) 10 mg/L phenol and (**b**) diluted acrylon wastewater. Reaction conditions are the same as those in Figure 6.

Two further experiments were conducted using 10 mg/L phenol and diluted acrylon wastewater as the pollutant for degradation. Figure 7a shows that, after 4 h of the TiO_2_-based photocatalytic degradation reaction, the TOC value of the phenol solution sharply decreased from its initial concentration of 8.03 gm/L to a final concentration of 1.65 gm/L; the mineralization yield of the 10 mg/L phenol can reach as high as 79%, which is much better than the methyl orange degradation, as mentioned above. However, when acrylon wastewater was used as the degradation target under the same experimental conditions, the mineralization yield was less than 50% (Figure 7b). As is known, phenol is normally hard to decompose due to its stable benzene ring structure; the results may illustrate that the TiO_2_ nanowire prepared using this method can be more effective in decomposing phenol than acrylon wastewater.

#### 2.3.2. PRD-Reactor Experiment

**Figure 8:**
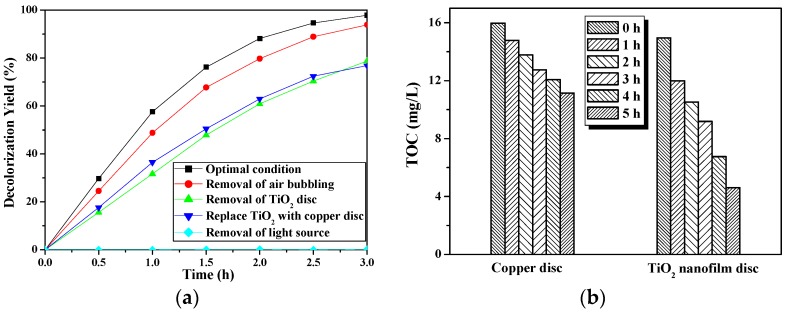
Photocatalytic activity tests of the photocatalytic rotating disc reactor (PRD-reactor). Reaction conditions: (**a**) 15 mg/L MO and (**b**) 20 mg/L phenol. Volume of degradation solution: 5 L; light source: 3 × 10 W UV lamp; photocatalyst: TiO_2_ nanowire deposited on 0.8 mm substrate with active area of 1248 cm^2^; disc rotating speed: 33 rpm. The optimal conditions meant that the experiment was conducted under all above-mentioned conditions, with air bubbling employed.

The influences of PRD-reactor parameters on reaction efficiency were studied using 15 mg/L MO as our degradation target. As shown in Figure 8a, under the optimal reaction conditions (black curve), the decolorization yield of MO almost reached 98% under UV lamp irradiation for 3 h with air bubbling being employed to enhance mixing and improve dissolved oxygen content. This result is used as a reference for evaluating the effects of each individual factor. First, the TiO_2_ disc was removed and air bubbling was maintained to understand the effect of the photolysis process on the degradation yield; the result (green curve) shows that the decolorization yield drops, but can still reach 79%, even without the TiO_2_ disc for photocatalytic reaction, which is attributed to the strong oxidization of UV photolysis. Air bubbling also increases the dissolved oxygen content in the solution, promoting the photolysis process. Subsequently, experiments with air bubbling removed and replacing the TiO_2_ disc with a copper (very stable under UV irradiation) disc, with and without disc rotation, were carried out to compare the effect of disc rotation, with and without air bubbling. In this case, the decolorization yield (blue curve) indicated that the disc rotation was advantageous, which can replace air bubbling and, hence, reduce costs; in addition, it can also benefit the photocatalytic reaction. Subsequently, air bubbling was removed from the optimal condition to further verify the influence of air bubbling (red curve), which slightly dropped the decolorization yield (93.9%) compared with the optimal case. Finally, the cyan curve obviously indicates that UV irradiation alone is the most essential factor in the entire PRD-reaction as the MO decolorization yield is almost 0% under the presence of the TiO_2_ disc and air bubbling, even including the adsorption of dyes on the surface of the TiO_2_ disc.

The photocatalytic degradation activity of the PRD-reactor for 20 mg/L phenol was also tested and results are shown in Figure 8b. The TOC value is used to characterize the phenol degradation rate. After 5 h of reaction, the TOC value of the phenol decreased from 14.95 mg/L to 4.61 mg/L, meaning a mineralization rate of 69%. However, the TOC removal rate of phenol was reduced to only 30% when the TiO_2_ nanowire disc was replaced with a copper disc, which indicates that the photocatalytic process plays a more important role than photolysis in the mineralization of this organic pollutant.

**Figure 9:**
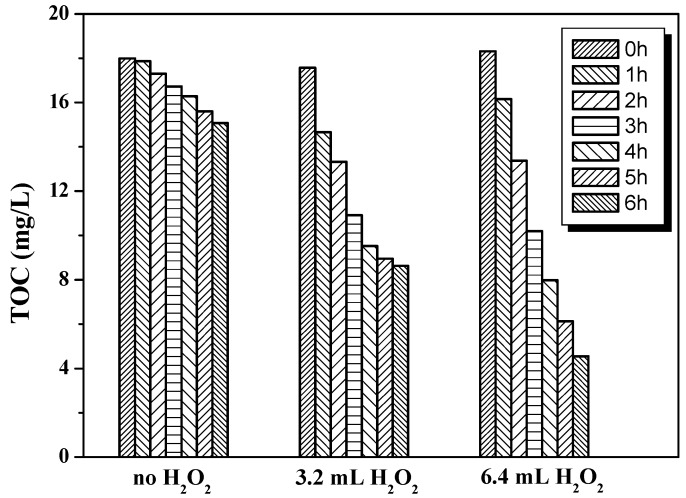
Photocatalytic activity test of the PRD-reactor for biodegraded wastewater.

Obviously, further decomposition of wastewater after the biodegradation process reaches a lower concentration (below 20 mg/L of TOC value) is difficult. Figure 9 shows the TOC values of the PRD-reactor treating biodegraded wastewater. As shown, the degradation effect of the pure PRD-reaction is not satisfactory for biodegraded wastewater, probably due to its rigid molecular structure. Therefore, in order to get a better reaction efficiency, a small dose of H_2_O_2_ (3.2~6.4 mL H_2_O_2_ in 5 L wastewater) was added as an enhanced oxidant in the reaction. The results show that the reaction activity can be greatly enhanced after the addition of H_2_O_2_. The TOC removal rate increased from 16.2% to 52% and 75.2% for 3.2 mL and 6.4 mL H_2_O_2_, respectively. More intensive research has been conducted to study the function of H_2_O_2_ in the advanced oxidation process. Here, the H_2_O_2_ was used as an electron acceptor in the photocatalytic reaction due to its higher activity than oxygen, in order to capture the photogenerated electrons to produce active OH• radicals, which can target pollutant molecules for an efficient degradation. Additionally, H_2_O_2_ reacted with photogenerated electrons could reduce the recombination of photogenerated electron-hole pairs, which could directly contribute to the degradation reaction.

**Figure 10:**
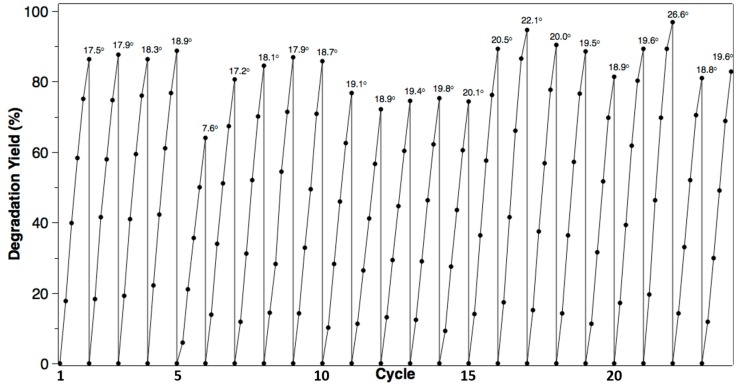
Repeatability tests with 20 mg/L phenol. The numbers above the curve correspond to the ambient temperature for each experiment; the interval between two adjacent points is 1 h.

Figure 10 shows the results of degradation using the TiO_2_ disc with a TiO_2_ nanowire film and was tested with 20 mg/L phenol in a consecutive cycle. As can be seen, after 23 cycles, the TiO_2_ nanowire film deposited on the Ti plate still maintained a good activity (>80%). The only influencing factor was temperature. The degradation yields could be similar under similar temperatures, and a higher temperature results in a higher yield. This shows that the degradation yield was affected by the temperature of the operation. As a whole, the result demonstrates a good repeatability for each cycle.

## 3. Discussion

**Figure 11:**
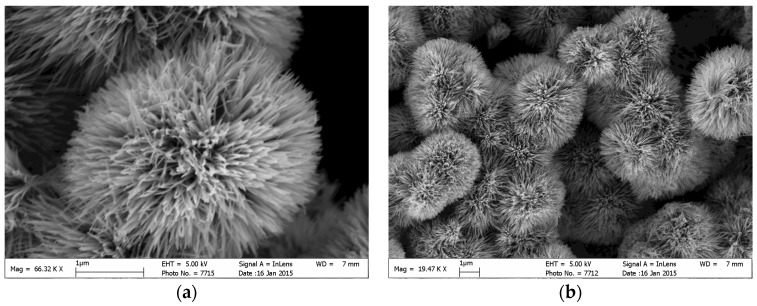

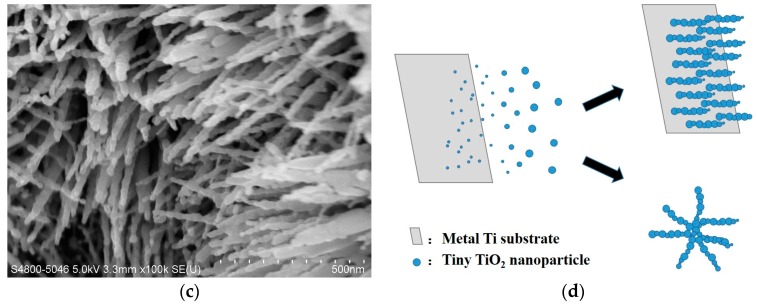
(**a**) SEM image of single TiO_2_ nanoflower; (**b**) SEM image of TiO_2_ powder collected from the post-depositional solution; (**c**) the clear SEM image of the branches of TiO_2_ nanoflower; (**d**) schematic diagram of the TiO_2_ deposition process.

Figure 11 shows the deposition process of the TiO_2_ nanostructure and the corresponding SEM diagrams at different magnifications. The deposition process of TiO_2_ nanowire arrays follow the steps listed below. First, metallic Ti was preliminarily oxidized to a Ti-ion state in the etching step, which triggered further oxidation in an acidic H_2_O_2_ solution to form TiO_2_ nanoparticles. In the deposition process, Ti-ion was dissolved in H_2_O_2_, then quickly oxidized to TiO_2_ and grown into tiny nanoparticles. According to the HR-TEM (Figure 1b) and SEM (Figure 11c) images, most of the tiny nanoparticles were deposited on the nearest metal Ti substrate to fabricate nanowire with a nanoflower structure under the appropriate H^+^ concentration, while the rest of the nanoparticles could not be immediately loaded, so they agglomerated with the growing point as the center to form a nanoflower via numerous nanowires in the solution (as shown in Figure 11a,b).

Despite the fact that TiO_2_ nanowire arrays deposited on a Ti plate can treat wastewater with considerable efficiency, the limitation of UV light absorption is still a barrier for TiO_2_ being applied for industrial purification. However, TiO_2_ nanowires composed of plenty of TiO_2_ nanoparticles led to a hierarchical structure and a high specific surface area, which could achieve a more effective doping with non-metal ions (such as C, N, B, P, etc.) to extend the light spectrum to visible light range. This can solve the limitation of TiO_2_ on light absorption, and widen its application under solar light irradiation.

## 4. Materials and Methods

### 4.1. Materials

Ti plates with 99.8% purity (Oudifu Metallic Material Ltd., Changsha, China) were used throughout the study. Major chemicals used included acetone (ACS, 99.9%, Merck, Kenilworth, NJ, USA), ethanol (ACS, 99.9%, Merck), HF (40%, Aladdin, Shanghai, China), HNO_3_ (65%, Sigma-Aldrich, St. Louis, MO, USA), H_2_O_2_ (30%, Aladdin, China), melamine, HCl (37%, Sigma-Aldrich), methy orange (ACS, AJAX, Seven Hills, NSW, Australia), Na_2_SO_4_ (AR, Aladdin, China), and phenol (ACS, Aladdin, China); all were used as received without further purification. All solutions were prepared with deionized water.

### 4.2. Preparation of TiO_2_ Nanowire Arrays

Cleaning: Rectangular titanium plates (20 mm × 30 mm) with three different thicknesses (i.e., 0.1, 0.2, and 0.8 mm) for the stagnant experiment. A circular titanium plate (φ200 mm in diameter) with a thickness of 0.8 mm was also prepared for the reactor experiment. All the Ti plates were ultrasonically washed in acetone, ethanol, and deionized water, consecutively, and dried at room temperature.

Etching: Cleaned Ti plates were immersed in a fluorine-containing solution, which consisted of HF, HNO_3_, and deionized water (1:3:6 volume) for 1 min; they were taken out and immediately ultrasonically cleaned with deionized water. Finally, they were dried at 60 °C for 30 min.

Deposition: After pretreatment, the small Ti plates (20 mm × 30 mm) were put into a well-mixed solution containing 15 mL of 30% H_2_O_2_ solution, 30 mg melamine, and 0.3 mL of 65% nitric acid. They were allowed to react at 80 °C for 72 h. The circular Ti plate was immersed in the prepared mixture solution at a total volume of 600 mL and allowed to react for 72 h at 80 °C.

Crystallization: After deposition, the as-prepared samples were rinsed with deionized water, and dried at 60 °C. The crystallization of TiO_2_ was achieved by annealing at 500 °C for 4 h under an atmospheric pressure.

### 4.3. Characterization

The samples of TiO_2_ nanowire arrays deposited on the Ti plate were characterized using field emission scanning electron microscopy (FESEM, Hitachi S-4800 and FESEM, LEO 1530). Transmission electron microscopy (TEM) observations were conducted with FEI Tecnai G2-20 S-TWIN scanning transmission electron microscope (STEM). X-ray diffraction (XRD) measurements were carried out in parallel mode using an X’Pert PRO diffractometer (PANalytical) with Cu Kα radiation (λ = 0.15406 nm), 2θ ranged from 20° to 80° with a step size of 0.02°/s. The operating tube voltage and current were 40 kV and 40 mA, respectively. The surface electronic states were analyzed using X-ray photoelectron spectroscopy (XPS, Perkin-Elmer PHI 5000). All the binding energy values were calibrated by using C1s = 284.6 eV as a reference. Ultraviolet-visible diffuse reflectance spectra (UV-vis DRS) were recorded using a Shimadzu UV 2600 spectrophotometer (Kyoto, Japan) with BaSO_4_ as a reference. Photoelectrochemical measurements were tested using an electrochemical station (CH Instruments Inc., CHI-660E, Bee Cave, TX, USA).

### 4.4. Major Design Parameters of the PRD-Reactor

**Figure 12:**
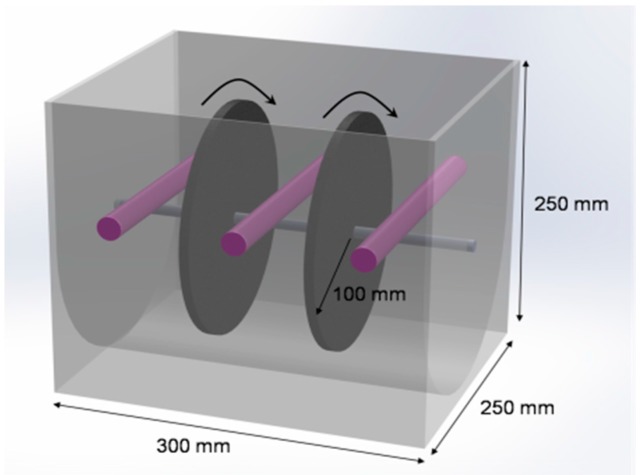
Configuration of the PRD-reactor.

As shown in Figure 12, the reactor is a rectangular tank with a working solution volume of ~5 L during operation. The rotating shaft is set at a height of 1 cm above the water surface. Three UV lamps were fixed on the wall of the reactor, which was positioned over the rotating shaft. The shaft across the reactor is located in the middle of reactor and was connected to an electric motor with a rotating speed that ranged from 1 to 40 rpm. The shaft holds two Ti plates with TiO_2_ nanowires deposited on its metal Ti surface; the distance between the lamp and the TiO_2_ photocatalyst was 5 cm. UV light sources with two power settings were used: 7 W for stagnant tests, and 10 W for PRD-reactor experiments. The total photocatalytic active area of the TiO_2_ was 1248 cm^2^.

## 5. Conclusions

In summary, a PRD-reactor, based on an immobilized TiO_2_ nanowire array film material, deposited on Ti plates, was designed for treating industrial wastewater with a high efficiency. The ordered TiO_2_ nanowire arrays of mixed phases (i.e., anatase and rutile) can be synthesized with a mild solvethermal method at 80 °C, which can simplify the industrial preparation of the photocatalyst. The advantage of this design is that the photocatalyst can be regenerated numerous times on the Ti plate. The as-obtained TiO_2_ exhibited excellent photocatalytic activity and repeatability for degrading representative water pollutants. The PRD-reactor that targeted industrial biodegraded wastewater could achieve a 75.2% TOC removal rate with the addition of trace amounts of H_2_O_2_ as a processing agent. In the stagnant experiment, the TOC removal rate for 10 mg/L phenol reached ~80% in 4 h.

In this study, the mineralization yield of the wastewater was the main assessment method achieved via TOC determination. For thorough wastewater purification, the antibacterial activity of photocatalytic process could also be considered in the assessment system in future studies. The novelty of the present study includes the following results:

1. Our PRD-reactor can be up-scaled for application in industrial wastewater treatment.
2. Capability of advanced treatment of biodegraded wastewater with a high efficiency.
